# Schizoaffective Disorder and Parkinson’s Disease: a case report

**DOI:** 10.1192/j.eurpsy.2024.1331

**Published:** 2024-08-27

**Authors:** A. Izquierdo De La Puente, P. del Sol Calderón, R. Fernandez Fernandez

**Affiliations:** ^1^Psychiatry, Hospital Universitario Puerta de Hierro de Majadahonda, Majadahonda; ^2^Psychiatry, Hospital Universitario Infanta Cristina, Parla, Spain

## Abstract

**Introduction:**

We present the case of a patient with schizoaffective disorder and Parkinson’s disease (PD), requiring treatment adjustment, with the use of high doses of quetiapine for the treatment of psychotic symptomatology.

**Objectives:**

The aim is to briefly review the treatment of dopaminergic psychosis in the elderly.

**Methods:**

Patient aged 86 years, institutionalised, presenting severe episodes of behavioural alteration, high anxiety and delusions of harm, together with auditory and visual hallucinations. As relevant physical history, the patient has AHT, aortic insufficiency, and bladder cancer operated on in 2012. As psychiatric history of interest, the patient has been diagnosed since his 30s with schizoaffective disorder, Parkinson’s disease and moderate-severe cognitive impairment secondary to the previous two.

As usual treatment, in addition to anticoagulation and antihypertensive therapy, the patient has been receiving L-dopa for his PD for years, antidepressant treatment with escitalopram 10mg, haloperidol 80 drops a day, divided into three doses, and lormetazepam 2mg as a hypnotic.

In addition to the symptoms described above, the patient had episodes of confusional features, as well as marked stiffness in the cogwheel and significant gait disturbance, having suffered several falls without serious repercussions.

**Results:**

Due to the comorbid neurological pathology, it was decided to progressively modify the treatment, withdrawing the benzodiazepine due to the risk of confusional disorder and replacing it with trazodone. Antipsychotic treatment was gradually replaced by extended-release quetiapine, reaching a maximum dose of 800mg. Likewise, escitalopram treatment is replaced by sertraline.

With this adjustment, there was an improvement in the psychotic symptoms, as well as in the anxious symptoms. Episodes of distress are NOT observed, and the patient’s functionality improves, allowing him/her to participate in daily activities, both cognitive stimulation and physiotherapy.

**Conclusions:**

The Spanish Society of Psychogeriatrics recommends that before using antipsychotics, it is advisable to first treat the underlying potentially treatable causes (pain, infections, toxic effects of drugs…), assess non-pharmacological interventions and always, if the use of antipsychotics is required, assess the risk-benefit ratio.

In relation to the above, it is not surprising that in the elderly, the use of second-generation antipsychotics is recommended in the first place, as opposed to the classical ones. The latter are only recommended in emergency situations where an almost immediate effect is required.

For dopaminergic psychosis, there are only controlled trials with clozapine. However, due to prescribing difficulties, aripiprazole or quetiapine is recommended in the first instance.

**Disclosure of Interest:**

None Declared

